# Comparison of accuracy of pediatric index of mortality-3 (PIM-3) and pediatric index of mortality-2 (PIM-2) scores in predicting outcome of children admitted to pediatric ICU of a tertiary care hospital

**DOI:** 10.12669/pjms.41.10.12061

**Published:** 2025-10

**Authors:** Minhaj Tabish, Muhammad Haroon Hamid, Rabiah Mahwish, Fizzah Haroon

**Affiliations:** 1 Minhaj Tabish Post-Graduate Resident, Department of Pediatric Medicine Unit-I, Mayo Hospital, Lahore, Pakistan; 2 Muhammad Haroon Hamid Chairman Department of Pediatric Medicine, King Edward Medical University (Mayo Hospital), Lahore, Pakistan; 3 Rabiah Mahwish Associate Professor (Community Medicine), Khawaja Safdar Medical College, Sialkot, Pakistan; 4 Fizzah Haroon, MBBS Student,Aga Khan University, Karachi, Pakistan

**Keywords:** Mortality, Accuracy, Pediatric, Calibration

## Abstract

**Objective:**

To compare the accuracy of pediatric index of mortality-3 and pediatric index of mortality-2 scores in predicting the mortality of children admitted to the pediatric intensive care unit of a tertiary care hospital in Pakistan.

**Methodology:**

This observational study conducted in the pediatric intensive care unit of Mayo Hospital, Lahore, Pakistan. In this study variables of pediatric index of Mortality-2 and pediatric index of Mortality-3 scores, along with baseline characteristics of children aged one month to 12 years, were recorded prospectively from 1^st^ May, 2024 to 31^st^ October, 2024. Sensitivity, specificity and positive and negative predictive values were determined for the two scores. The standardized mortality rate was calculated to compare the observed and predicted mortality of two scores. Area under the Receiver Operating Characteristic (AU-ROC) curves and Hosmer-Lemeshow Goodness-of-fit tests were used to determine which model had better discrimination and calibration.

**Results:**

A total of 188 patients (57.5% males, 42.5% females) with a median age of 10 months (Interquartile range: 4-36 months) were included in the study. The sensitivity of pediatric index of Mortality-2 and pediatric index of Mortality-3 was 85.71 % and 80.35%, respectively, while the specificity was 73.48% and 82.57%, respectively. The standardized mortality rate of pediatric index of Mortality-2 and pediatric index of Mortality-3 scores were 0.67 and 0.82, respectively. Both scores had good discrimination, while pediatric index of Mortality-3 performed better with area under the Receiver Operating Characteristic curve of 0.858 compared to pediatric index of Mortality-2, which was 0.842. For both tests, the Hosmer-Lemeshow goodness-of-fit tests showed good calibration (p > 0.05).

**Conclusion:**

The predictive accuracy of the pediatric index of Mortality-3 score was better than that of the pediatric index of Mortality-2 score, although both scores performed well and can be used for mortality prediction.

## INTRODUCTION

Mortality is among the most frequently assessed health outcomes in a Pediatric Intensive Care Unit (PICU). The cardinal aim of a PICU is to provide quality care, which is achieved by intensively monitoring and treating sick patients who are at high risk for mortality.^1^ To assess the performance of an ICU, many tools have been designed in terms of mortality prediction scores like Pediatric Risk of Mortality (PRISM, PRISM-3), Pediatric index of mortality (PIM, PIM-2, PIM-3), etc.

The PIM score was developed because models like the PRISM-3, which use data collected during the first 12 or 24 hours after admission, might be affected by the quality of the initial ICU management, which could, in turn, affect predicted mortality.^2^,^3^ Initially, the PIM score was developed using data from PICUs in Australia and the United Kingdom (UK).^3^ PIM was updated to PIM-2 using data from Australia, New Zealand, and the UK.^4^ In 2013, Straney et al. developed a PIM-3 model based on more recent data that provides better mortality estimation.^5^ PIM-3 underwent several modifications in the ‘post-procedural recovery’ and ‘risk indicator’ fields.^5^ Other mathematical adjustments were performed for variables like systemic blood pressure, base deficit, and partial pressure of arterial oxygen (PaO_2_) to Fraction of inspired oxygen (FiO_2_) ratio (PaO_2_/FiO_2_). These updates improved mortality risk estimation.^5^

Validation and comparative studies of these tools have been done in many countries. A comparative study on PRISM-III, PIM-2, and PIM-3 in an Indian setting showed better discrimination and ease of data collection of PIM-2 and PIM-3 models.^2^ A study conducted in Colombia reported PIM-3 as a better tool than PIM-2;^6^ a similar finding was reported by Jung et al.^7^ Previously, a comparative study was done in Pakistan, favoring PIM-2 over PRISM and PELOD.^8^ Another study validating the PIM-2 score was conducted in our hospital, reporting sensitivity, specificity, positive predictive value, and negative predictive value of 54.3%, 83.3%, 58.1%, and 81.1%, respectively.^9^

So far, only a few studies have validated PIM-3, and even fewer from developing countries.^2^,^10^ No comparative study of PIM-2 and PIM-3 has been conducted in Pakistan. Pakistan’s population characteristics are much different from those in Western countries. We have more admissions with malnutrition, critically sick patients who report late to the hospital, and less advanced and understaffed PICUs. Furthermore, the PIM-2 and PIM-3 models were developed and validated in mixed ICU settings with mixed medical and surgical patients. In contrast, post-surgical patients are not routinely admitted to our PICU. As a result, some of the variables are occasionally accounted for in score calculation.

Due to these peculiar circumstances, this study was conducted to compare the accuracy of PIM-3 and PIM-2 scores in predicting the mortality. This may help identify an effective model for mortality prediction in our setting.

## METHODOLOGY

This observational study was conducted in our nineteen-bed tertiary care PICU from 1^st^ May, 2024 to 31^st^ October, 2024 after approval of synopsis,. All children aged one month to 12 years were enrolled. Patients who died within the first eight hours or were discharged or shifted out of PICU within 24 hours of admission were excluded. A sample size of 188 patients was calculated with a 90% confidence interval and a margin of error of 6%, assuming the prevalence of mortality in the enrolled population to be 29.9%.^9^ Consecutively admitted patients whose parents gave informed consent were enrolled in the study. Variables were collected within the first hour of admission along with demographic data, total duration of PICU and hospital stay, and outcome. Compared to PIM-2, PIM-3 features some notable adjustments. First, PaO_2_/FiO_2_ is given a score of 0.23 if either or both values are unknown. Second, PIM-3 has three risk categories: very high-risk, high-risk and low-risk, instead of two (high-risk and low-risk). Third, a new field has been added to the ‘post-procedural recovery’ variable.^5^ Details of PIM-2 and PIM-3 are in the supplementary file.

### Ethical approval:

It was obtained from the Institutional Ethic Committee (Letter No. 498/RC/KEMU; Dated: May 23, 2022).

### Statistical analysis:

Data was analyzed using SPSS version 20 (Statistical Program for Social Sciences). Descriptive statistics were used to report age, gender, malnutrition, length of stay in the PICU and the hospital, diagnosis and outcome. The normality of the data was assessed using the Kolmogorov-Smirnov test. The sensitivity, specificity and positive and negative predictive values of both scores were determined. Standardized Mortality Rate (SMR) was calculated as the ratio of observed to predicted mortality for the two scores. The accuracy of the two scores was evaluated by plotting receiver operating characteristic (ROC) curves, with discrimination assessed by the area under the curve (AU-ROC). The calibration of the models was evaluated by the Hosmer-Lemeshow goodness-of-fit test.

## RESULTS

A total of 188 patients (57.5% males, 42.5% females) were enrolled in the study. The median age was 10 months (interquartile range [IQR]: 4-36 months), and the median weight was six kg (IQR: 4-11 kg). Mortality observed was 29.78% (n=56). The median duration of ICU stay was three days (IQR: 2-4 days), while the median duration of hospital stay was five days (IQR: 4-9 days). The most frequent diagnoses were pneumonia (54, 29%), meningitis/meningoencephalitis (34, 18%), and diarrhea with sepsis (26, 14%), while 99 (52%) patients were malnourished. Among the malnourished, those with moderate malnutrition were 25 (25%), and those with severe malnutrition were 74 (75%). Patients who needed assisted ventilation were 161 (85%) (Table-I).

**Table-I:** Baseline demographic characteristics (N=188).

Variable	Total n (%)	Mortality n (%)	p-value
***Diagnosis***			0.01
Respiratory Diseases	72 (38)	16 (22)	
Neurological Diseases	53 (28)	15 (28)	
GI/Hepatobiliary Diseases	33 (17.5)	10 (30)	
Sepsis	9 (5)	7 (78)	
Cardiac diseases	5 (3)	3 (60)	
Others	16 (8.5)	5 (31)
***Age (months)***			0.19
<60 months	154 (82)	49 (32)	
>60 months	34 (18)	7 (20)
***Gender***			0.09
Male	108 (57.5)	27 (25)	
Female	80 (42.5)	29 (36)	
Malnourished	99 (52)	34 (34)	0.10
Mechanical Ventilation	161 (85)	54 (33)	0.006

**Fig.1:**
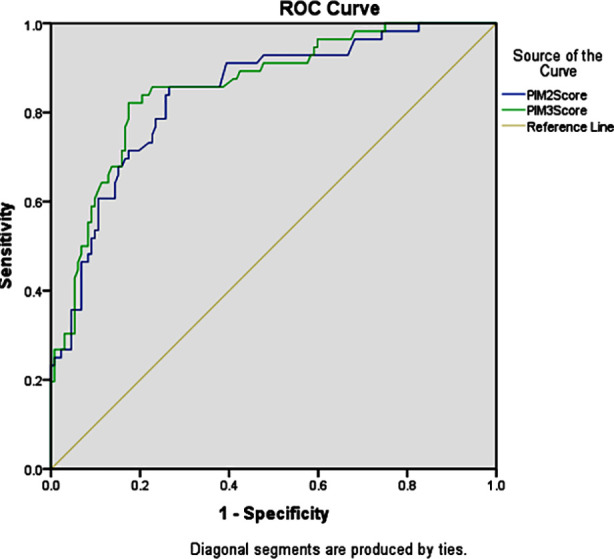
ROC Curve.

The sensitivity and specificity of PIM-2 were 85.7% and 73.4%, respectively and those of PIM-3 were 80.3% and 82.5%. The SMR of PIM-2 and PIM-3 was 0.67 and 0.82, respectively. The AU-ROC of PIM-2 and PIM-3 was 0.842 (95% CI: 0.780-0.903) and 0.858 (95% CI: 0.799-0.916), respectively. (Table-II, Fig.1).

**Table-II:** Comparison of PIM-2 and PIM-3 models (N=188).

Statistical parameter	PIM-2	PIM-3
Sensitivity	85.71	80.35
Specificity	73.48	82.57
Positive Predictive Value (PPV)	57.83	66.17
Negative Predictive Value (NPV)	92.38	90.83
AU-ROC	0.842	0.858
Hosmer-Lemeshow x^2^ (p-value)	4.86 (0.77)	7.53 (0.48)
SMR	0.67	0.82

## DISCUSSION

The sensitivity of PIM-2 (85.71%) was better, while the specificity of PIM-3 (82.57%) was better as compared to PIM-2. The mortality rate was 29.78%. Both PIM-2 and PIM-3 over-predicted mortality, with SMR being 0.67 and 0.82, respectively. SMR of PIM-3 being closer to one showed that PIM-3 was better at estimating mortality risk than PIM-2. The discriminatory power was evaluated by determining the value of AU-ROC. A value of 0.90 or more is considered excellent, 0.80-0.89 is good and 0.70-0.79 is fair or acceptable. The AU-ROC of PIM-3 was 0.858 (95% CI: 0.799-0.916), showing better discrimination ability than PIM-2, which was 0.842 (95% CI: 0.780-0.903). While PIM-3 outperformed PIM-2, the predictive accuracy of both models was good (Table-III). Calibration was tested using the Hosmer-Lemeshow goodness-of-fit test, with a p-value greater than 0.05 indicating good calibration. Our study reported good calibration (p>0.05) of both models.

**Table-III:** Area under the ROC curve.

Area under the ROC				
Test Result Variable(s)	Area	Std. Error	Asymptotic 95% Confidence Interval	
			Lower bound	Upper Bound
PIM-2 Score	.842	.031	.780	.903
PIM-3 Score	.858	.030	.799	.916

In Pakistani settings, one study showed an SMR of PIM-2 to be 1.4, indicating under-prediction, while another study by Mazhar et al. reported an SMR of 1.07, which was closer to one.^8^,^9^ Studies from developed countries have shown SMR less than one, similar to our study.^4^,^7^,^11^ A similar result is seen in a Colombian study reporting SMR of 0.66 for PIM-2 and 1.00 for PIM-3.^6^ Jung et al. also reported SMR closer to one (1.11) for PIM-3 as compared to PIM-2 (0.84).^7^ Similarly, a study from Italy reported SMR of 0.98 for PIM-3 and 0.80 for PIM-2.^11^ SMR closer to one indicates better performance of a model. SMR provides the standardization of mortality rate because when measuring crude mortality, variables like disease severity, case-mix, and co-morbidities are not considered. SMR is a standard and also a benchmark for quality measures.

This study showed good discrimination for both scores, which indicates good performance of both models. A study from Pakistan also reported good discrimination (AU-ROC of 0.88)^8^ while another study from Pakistan by Mazhar et al. demonstrated fair discrimination of PIM-2.^9^ PIM-3 performed better than PIM-2 in our study and in other studies as well.^7^,^11^,^12^ Malhotra et al. reported fair discrimination of PIM-3, Ramazani et al. and Wong et al. reported good discrimination, while studies from Saudi Arabia and Egypt reported excellent discrimination (0.93 and 0.97 respectively) of PIM-3.^13^-^17^ This study and studies from other countries have reported PIM-3 to be a better scoring model than PIM-2 and the reason seems to be the fact that PIM-3 has addition of new variables and adjustments that makes it an updated model.

Our study reported good calibration (p>0.05) of both models, consistent with several other studies.^2^,^7^ However, several studies from Pakistan and India showed poor calibration of PIM-2 and PIM-3, respectively.^9^,^18^ Similarly, Sankar et al. also reported poor calibration of both models.^12^ Validation studies from the developing countries have shown mixed results for calibration, while good calibration was seen in the countries where these models were tested. Poor calibration can be due to several factors that may have caused higher observed mortality than expected mortality. Factors like limited resources, disease severity, case-mix, and failure of scoring systems to predict the actual situation could be the reasons for poor calibration in the developing countries.^8^

The mortality rate was 29.78%, similar to studies from Pakistan^8^,^9^ and other developing countries^2^,^12^,^19^ but much higher than in developed countries.^4^,^7^,^11^,^13^,^20^ Diagnoses were also comparable, the most common being pneumonia, diarrhea, meningitis and sepsis.^9^ Surgical/post-surgical patients were not admitted, unlike studies from developed countries.^4^,^5^ Our high mortality rate could be due to the majority of admissions of critically ill patients and referrals from remote areas with limited healthcare facilities. A large number of patients (85%) needed assisted ventilation and 31% were intubated during the first hour of admission. Sick patients with a lack of pupillary response (3%) were also admitted. Patients admitted with ‘very high-risk’ diagnosis were 9 (4.7%) and only one out of them survived. These ‘very high-risk’ diagnoses were cardiac arrest, acute liver failure, lymphoma and immunodeficiency.

PICUs in low-middle-income countries (LMICs) like ours have limited resources. Therefore, the high burden of complicated cases, delayed presentation, and limited resources have made intensive care a challenging field. Providing adequately trained staff, necessary medications/equipment, supportive leadership, a conducive environment and frequent training programs empowers healthcare providers and significantly reduce morbidity and mortality.^21^ Mortality prediction tools/scoring systems help improve resource allocation and ensure timely PICU transfers.^22^ Early identification and prompt decision-making help escalate respiratory/cardiovascular support wherever needed and thus ensure effective management.^22^

Although our PICU admitted only non-surgical patients, both models demonstrated good accuracy, with PIM-3 outperforming PIM-2. Therefore, implementing PIM-3 in our PICUs would be equally accurate even without surgical variables of the score. We recommend adopting PIM-3 over PIM-2 as a contemporary instrument for mortality prediction and benchmarking the quality of care.

### Limitations:

First, it was a single-center study; thus, the findings may not be generalizable to the entire pediatric population of Pakistan. Second, surgical variables were not accounted in for score calculation because surgical/post-surgical patients were not admitted to our PICU. A multi-center study involving surgical and medical PICUs needs to be conducted in Pakistan.

## CONCLUSION

Although both scores performed well and can be used for mortality prediction, PIM-3 was superior to PIM-2 in terms of its predictive accuracy.

### Declarations:

This study does not involve personal, financial, or other conflicts of interest. Grammarly was used to check the grammar. No other AI tool was used in the writing of this article.

### Authors’ Contribution:

**MT:** Conception & design of research; data collection and analysis; draft of manuscript; finalization of manuscript; ensured data accuracy and responsible/accountable for data accuracy and integrity of the work.

**MHH:** Conception of work; helped with interpretation of data; drafting and critical review of manuscript; final approval of manuscript with other authors, ensuring accuracy of information.

**RM:** Helped in Data analysis & interpretation; critical review of the manuscript; approval of the manuscript.

**FH:** Helped in interpretation of results, critically review the final draft; review of the literature & literature search.
